# Identification of a novel *LPL* nonsense variant and further insights into the complex etiology and expression of hypertriglyceridemia-induced acute pancreatitis

**DOI:** 10.1186/s12944-020-01249-z

**Published:** 2020-04-07

**Authors:** Xiao-Yao Li, Na Pu, Wei-Wei Chen, Xiao-Lei Shi, Guo-fu Zhang, Lu Ke, Bo Ye, Zhi-Hui Tong, Yu-Hui Wang, George Liu, Jian-Min Chen, Qi Yang, Wei-Qin Li, Jie-Shou Li

**Affiliations:** 1 Surgical Intensive Care Unit (SICU), Department of General Surgery, Jinling Hospital, Medical School of Nanjing University, Nanjing, China; 2 Department of Intensive Care Unit, The Affiliated Drum Tower Hospital, Medical School of Nanjing University, Nanjing, China; 3 Department of Gastroenterology, Subei People’s Hospital, Clinical Medical College of Yangzhou University, Yangzhou, China; 4 Key laboratory of Molecular Cardiovascular Sciences, Ministry of Education, Institute of Cardiovascular Sciences, Health Science Center, Peking University, Beijing, China; 5 Inserm, EFS, University of Brest, UMR 1078, GGB, F-29200 Brest, France

**Keywords:** Gene-environment interaction, Genotype and phenotype relationship, Hypertriglyceridemia-induced acute pancreatitis, Lipoprotein lipase, *LPL* gene, Nonsense variant, Triglyceride

## Abstract

**Background:**

Hypertriglyceridemia (HTG) is a leading cause of acute pancreatitis. HTG can be caused by either primary (genetic) or secondary etiological factors, and there is increasing appreciation of the interplay between the two kinds of factors in causing severe HTG.

**Objectives:**

The main aim of this study was to identify the genetic basis of hypertriglyceridemia-induced acute pancreatitis (HTG-AP) in a Chinese family with three affected members (the proband, his mother and older sister).

**Methods:**

The entire coding and flanking sequences of *LPL*, *APOC2*, *APOA5*, *GPIHBP1* and *LMF1* genes were analyzed by Sanger sequencing. The newly identified *LPL* nonsense variant was subjected to functional analysis by means of transfection into HEK-293 T cells followed by Western blot and activity assays. Previously reported pathogenic *LPL* nonsense variants were collated and compared with respect to genotype and phenotype relationship.

**Results:**

We identified a novel nonsense variant, p.Gln118* (c.351C > T), in the *LPL* gene, which co-segregated with HTG-AP in the Chinese family. We provided in vitro evidence that this variant resulted in a complete functional loss of the affected *LPL* allele. We highlighted a role of alcohol abuse in modifying the clinical expression of the disease in the proband. Additionally, our survey of 12 previously reported pathogenic *LPL* nonsense variants (in 20 carriers) revealed that neither serum triglyceride levels nor occurrence of HTG-AP was distinguishable among the three carrier groups, namely, simple homozygotes, compound heterozygotes and simple heterozygotes.

**Conclusions:**

Our findings, taken together, generated new insights into the complex etiology and expression of HTG-AP.

## Introduction

Acute pancreatitis (AP) is an acute inflammatory disease that is characterized by local pancreatic inflammation and consequently systemic inflammatory response [1, 2]. Gallstones, alcohol abuse and massive hypertriglyceridemia (HTG) are generally thought to be three leading etiologies of AP worldwide [3]. However, unlike in Western countries, HTG, rather than alcohol abuse, is the second leading cause of AP in China [4]. Hypertriglyceridemia-induced acute pancreatitis (HTG-AP) is defined by serum triglyceride (TG) level exceeding 11.3 mmol/L (1000 mg/dL) or between 5.6 to 11.3 mmol/L (500~1000 mg/dL) together with lipemic serum [5, 6]. As compared to other etiologies, HTG-AP is usually more severe and has higher recurrence rate [7, 8].

According to the etiology, HTG can be divided into primary and secondary HTG. Secondary HTG is usually caused by metabolic syndrome, diabetes, alcohol consumption, obesity, chronic renal failure, etc. [9] Primary HTG is caused by genes defects related with TG metabolism, including lipoprotein lipase (*LPL*), apolipoprotein C-II (*APOC2*), apolipoprotein A-V (*APOA5*), glycosylphosphatidylinositol-anchored high density lipoprotein-binding protein 1 (*GPIHBP1*) and lipase maturation factor 1 (*LMF1*). LPL is the key enzyme that catabolizes TG in non-hepatic tissues [10]. APOC2 and APOA5 act as essential LPL activators [11, 12]. LMF1 is involved in the folding and expression of LPL [13]. GPIHBP1 mediates the transmembrane transport and binding of LPL [14].

However, in most cases, the cause of HTG is complex [15]. Severe HTG was recently shown to be primarily polygenic [16], and there is increasing appreciation of the interplay between primary and secondary etiological factors in causing severe HTG [17, 18]. In this study, we reported a novel *LPL* nonsense variant in one typical Chinese family with HTG-AP history and discussed insights into the complex etiology of HTG-AP gleaned from the so far reported pathogenic *LPL* nonsense variants.

## Methods

### Ethical statement

This study was approved by the Ethics Committee of Jinling Hospital. Informed consent was obtained from all participants.

### Family description

The male proband had been suffered from recurrent severe HTG-AP since 26 years old, respectively in 2003, 2007, 2014 and 2017. He has had hypertension for 7 years and abused alcohol for more than 5 years (250–350 g/d). His body mass index (BMI) was normal (22.7 kg/m^2^). His mother and older sister also respectively had one- and two-times onset of HTG-AP.

### Sequencing of the *LPL*, *APOC2*, *APOA5*, *GPIHBP1* and *LMF1* genes

Genomic DNA was extracted from blood by the Gentra Puregene Blood kit (Qiagen, Dusseldorf, Germany) according to the manufacturer’s instructions. All exons and exon/intron boundaries of the *LPL*, *APOA5*, *APOC2*, *LMF1* and *GPIHBP1* genes were analyzed by sanger sequencing [18].

### Population allele frequency reference and variant nomenclature

Population allele frequencies of variants found in this study were evaluated using the Genome Aggregation Database (gnomAD) genome dataset [19] via VarSome [20]. Variant nomenclature was in accordance with Human Genome Variation Society (HGVS) recommendations [21]. NM_000237.3 was used as the *LPL* mRNA reference sequence.

### Plasma lipid profile analysis

Blood samples were taken from the proband after fasting for 12 h. Serum TG, TC, HDL, LDL levels were measured enzymatically on an automatic analyzer (Hitachi High-Tech, 7600–120, Japan).

### Post-heparin LPL mass analysis

Post-heparin blood samples were collected into Na-EDTA tubes 10 min after intravenous heparin injection (60 IU/kg body weight) and fasting for 12 h. Post-heparin plasma LPL mass was detected by immunoassay using the Human LPL Elisa kit (TSZ Biological Trade, USA).

### LPL activity analysis

LPL activity was in principle measured through detecting free fatty acid (FFA) concentration [22]. The reaction substrate, termed buffer A, was composed of 1 ml TG-rich serum (TG concentration, > 3000 mg/dL) from *Gpihbp1*-deficient mice (*Gpihbp1*^−/−^) [23], 0.18 g 10% fatty acid-poor bovine serum albumin (BSA) (Miles, West Haven, CT), 0.031 mg heparin, 0.012 g NaCl and 0.3 mmol Tris-HCl Buffer (pH 8.5), in a final volume of 5 mL. 5 μL buffer A were mixed with 5 μL serum from wild-type rats and 5 μL test sample, and incubated at 37 °C for 60 min. [Note that serum from either *Gpihbp1*-deficient mice or wild-type rats was pre-incubated for 10 min at 62.5 °C in order to inactivate any residual endogenous lipase activity.] FFA concentration was determined in triplicate on a spectrophotometer (Thermo Multiskan GO) using the Wako kit, NEFA-HR(2).

In the case of human serum test sample, the FFA concentration represented the total post-heparin lipase activities that comprised LPL and hepatic lipase (HL) activities. To correct for the contribution from HL, 1 M NaCl was added and incubated for 60 min, so that the LPL activity can be completely inhibited [24]. LPL activity was then calculated by the difference between total post-heparin lipase activity and HL activity. All assays were performed in triplicate.

### Plasmid construction and transfection

Human wild-type and c.352C > T mutant *LPL* coding sequences were synthesized and cloned into pcDNA3.1 (Vigene Biosciences), respectively. Sequence accuracy of the inserts was confirmed by Sanger sequencing.

HEK-293 T cells (ATCC, CRL-3216) were cultured in Dulbecco’s Modified Eagle’s Medium (DMEM, high glucose from Lonza, C11995500BT) containing 10% Fetal Bovine Serum (FBS) and 1% penicillin-streptomycin. Plasmids (1.5 μg/mL) were transiently transfected into HEK-293 T cells using Lipofectamine 3000 (Thermo, L3000015) in 6-well plates (Costar, 3516) according to the manufacturer’s instructions. After 6 h, the cells were changed into DMEM medium with 2% FBS. After 48 h, cells and medium were harvested separately. Proteins were extracted from cells solubilized in 70 μL RIPA (Beyotime, P0013E) with 7 μL protease inhibitor (PI, Roche, 4,693,116,001), and stored at − 20 °C. Protein concentration was determined by the BCA method. Cell medium was collected after inhibition with 20 U/mL heparin-DMEM (0.5 ml DMEM and 8 μL heparin (20 units/mL) for each well) for 30 min, centrifuged at 1000 r/min for 5 min, and the supernatant was stored at − 20 °C.

### Western blot analysis

Cell proteins were mixed with SDS-PAGE Protein Loading Buffer and incubated at 95 °C for 5 min. Proteins were size-separated by SDS-PAGE (10% acrylamide gel, 130 V, 90 min), transferred onto a nitrocellulose membrane (220 mA, 120 min), blocked for 1 h with 5% BSA, and washed 3 times for 15 min with 0.2% Tris-Buffered Saline with Tween 20 (TBS-Tween). Membranes were incubated overnight with primary antibodies, washed 3 times for 15 min with 0.2% TBS-Tween, incubated for 1 h with HRP-conjugated secondary antibodies, and washed 3 times for 15 min with 0.2% TBS-Tween. After 5-min incubation with chemiluminescent HRP substrate (Thermo Scientific), bands were visualized by Chemidoc XRS System (Clinx Science Instruments, Shanghai, China) and analyzed by Image Lab Software (Clinx Science Instruments, Shanghai, China). The antibodies used were mouse anti-LPL (Santa, sc-73,646) (1:200 dilution), rabbit anti-GAPDH (Santa, sc-69,778) (1:2000 dilution), goat anti-rabbit IgG H&L (HRP) (Abcam, ab6721) (1:10000 dilution), and rabbit anti-mouse IgG H&L (HRP) (Abcam, ab6728) (1:5000 dilution).

### Collation of previously published pathogenic *LPL* nonsense variants

Key words including “lipoprotein lipase”, “mutation”, “nonsense” and “termination” were used for searching previously reported pathogenic *LPL* nonsense variants in PubMed.

## Results

### Clinical findings and treatment of the proband

**Fig. 1:**
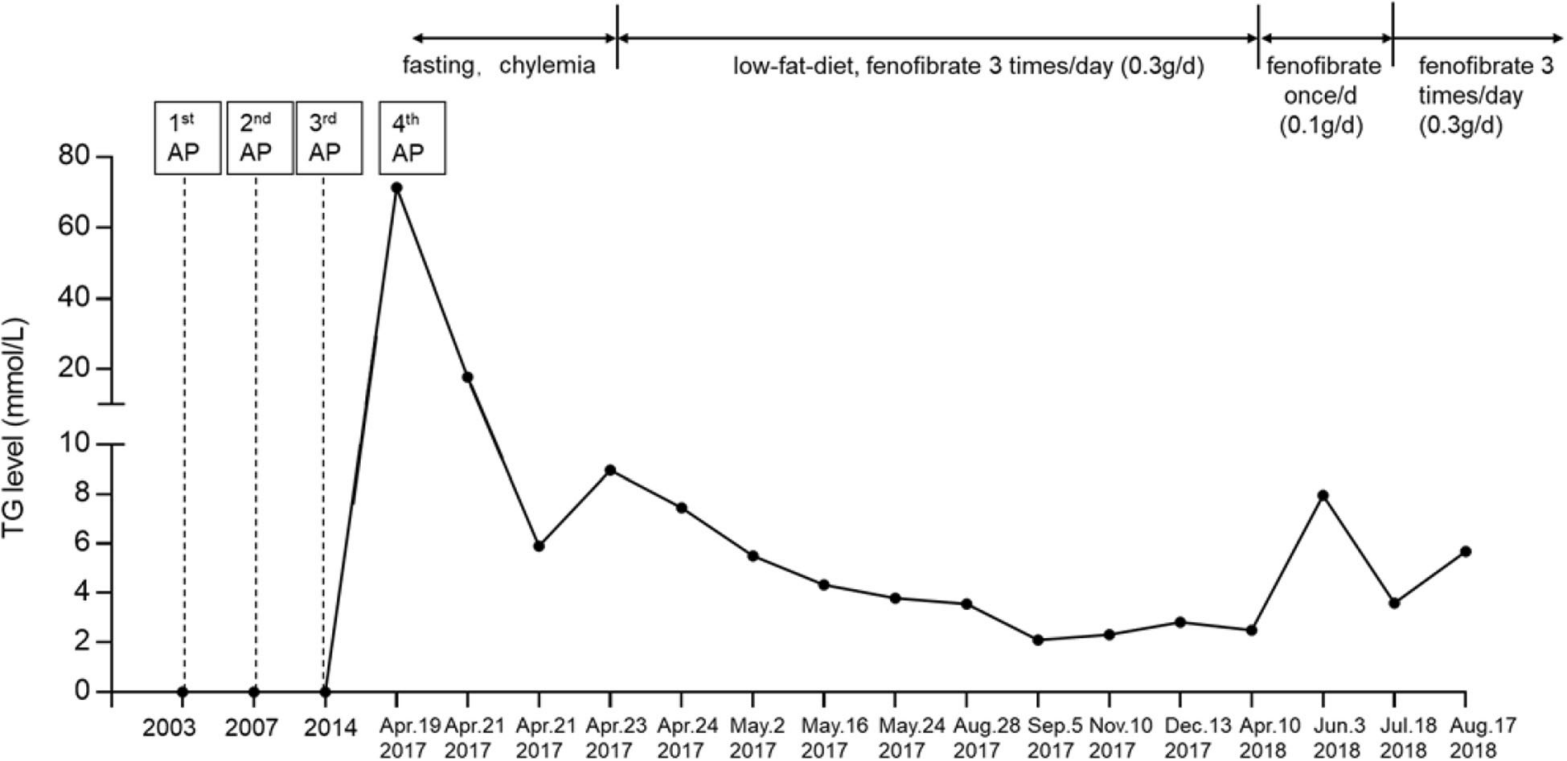
A summary of the patient’s disease history and his TG levels and treatments since the fourth attack of AP. TG, triglyceride; AP, acute pancreatitis

**Fig. 2:**
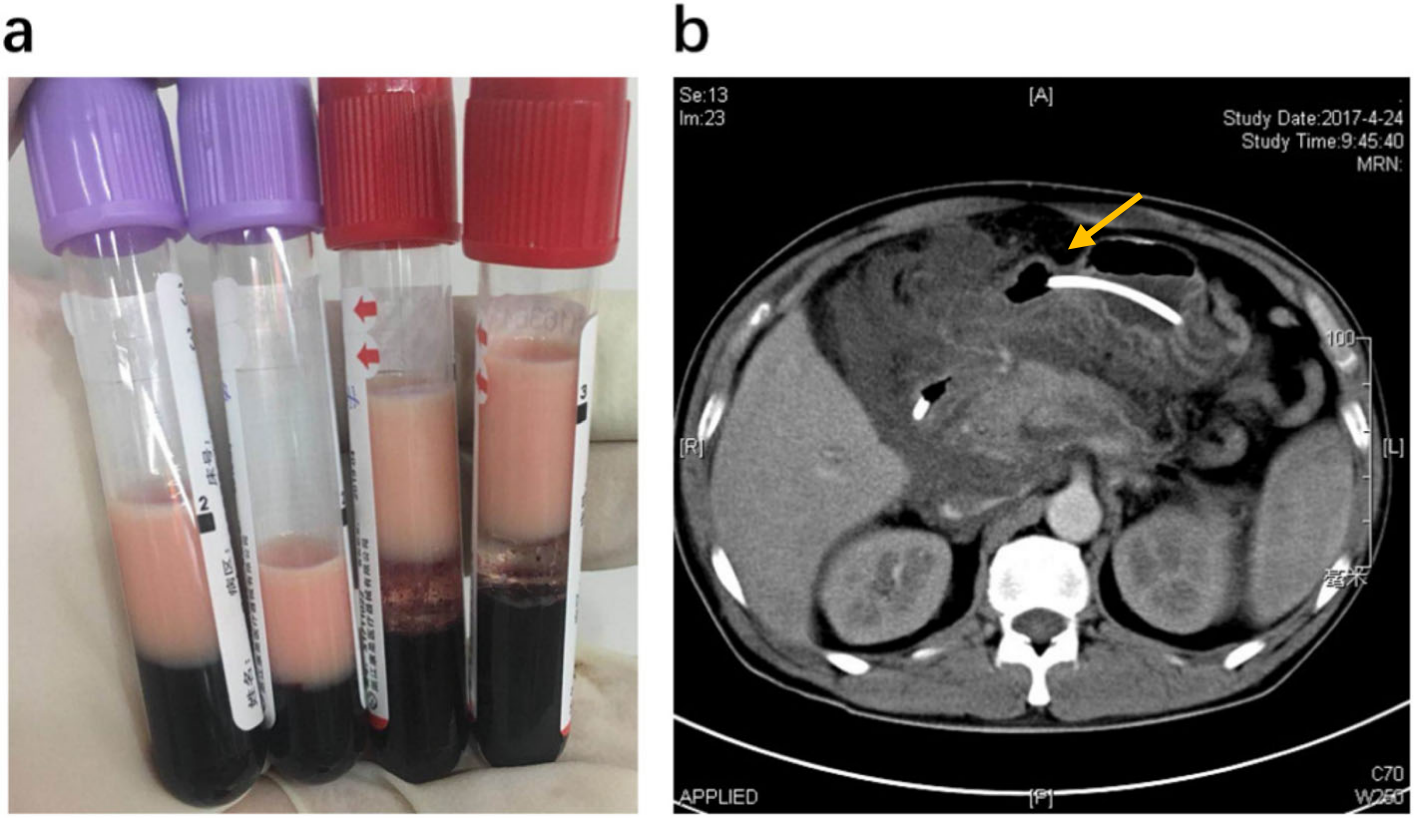
Two clinical observations of the patient upon admission to our service. **a** Blood samples showing chylemia. **b** Abdominal computed tomography showing enlarged pancreas with adjacent water density shadow and disappearance of the space between the pancreas and peripancreatic adipose tissues (arrow)

At his latest bout of AP in 2017, the proband was transferred into our severe acute pancreatitis therapy center in Jinling Hospital. His TG level was 71.3 mmol/L (6313.3 mg/dL) (Fig. 1) and his plasma was milky (Fig. 2a), fulfilling the definition of extreme HTG [25]. Physical examination revealed epigastric tenderness without rebound tenderness or Murphy’s sign. Laboratory examination revealed elevations in amylase level (446 U/L), white blood cell count (19.86 × 10^9^/L), inflammation biomarkers CRP (263.4 mg/L), IL-6 (121.6 ng/L) and PCT (2.93 μg/L), and renal function biomarkers CRE (289 μmol/L) and BUN (13.3 mmol/L). Abdominal computed tomography showed evidence of AP (Fig. 2b). Moderate-severe AP was diagnosed in accordance with the 2012 revision of Atlanta classification [5].

The proband was treated with enzyme inhibitors, anti-infection agents and fasting. His TG level decreased rapidly to 17.8 mmol/L (1576.19 mg/dL) 2 days later, and was determined to be 5.5 mmol/L (487.05 mg/dL) when discharged on May 2nd, 2017. During his hospitalization, the patient well tolerated enteral nutrition and adhered to a low-fat diet plus the lipid-lowering drug fenofibrate (0.3 g/d).

In most of the follow-up period, the patient has taken 0.3 g/d fenofibrate, low-fat diet, dry out and exercise (1 h /day), keeping the TG level within the mild to moderate range (defined as 2~9.9 mmol/L in accordance with Dron et al. [16]). However, once he took 0.1 g/d fenofibrate, an obvious increase in TG level was observed (Fig. 1).

### Genetic findings

**Fig. 3:**
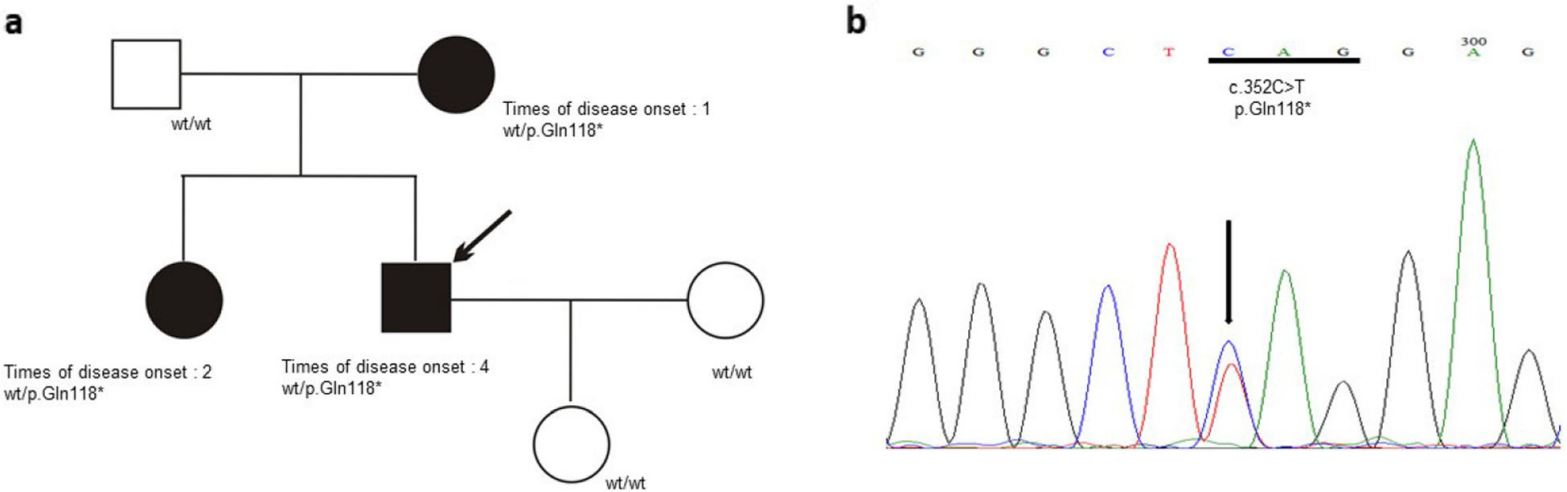
Identification of a novel heterozygous nonsense variant in the *LPL* gene. **a** Family pedigree. Arrow indicates the proband. Patients with hypertriglyceridemia-induced acute pancreatitis are indicated by black symbols whilst clinically unaffected family members are indicated by empty symbols. *LPL* genotypes are provided for all subjects. wt, wild-type. **b** Sanger sequencing electropherogram showing the heterozygous C > T single nucleotide substitution at position c.352 of the *LPL* gene (indicated by arrow) that would change the codon for glutamine at position p.118 (underlined) to a stop codon (i.e., p.Gln118*)

Sequencing of the *LPL*, *APOA5*, *APOC2*, *LMF1* and *GPIHBP1* genes in the proband detected four gene variants, as one *LPL* nonsense variant (Fig. 3) and three *LMF1* synonymous variants (Supplemental. Fig. S1). All three *LMF1* synonymous variants are common in the general populations (Table 1) and therefore were excluded from further consideration. The *LPL* nonsense variant, p.Gln118* (c.351C > T), is absent from the gnomAD database (Table 1). The LPL p.Gln118*was also detected in the proband’s mother and sister (Fig. 3a), but not in others 256 unrelated HTG-AP patients. Additionally, the *LPL* p.Gln118* nonsense variant has not previously been reported.

**Table 1:** Variants found in the present study

Gene	mRNA reference	Variant		Allele frequency in gnomAD genome dataset		rs number
		Nucleotide change	Amino acid change	East Asian population	All populations	
*LPL*	NM_000237.3	c.352C > T	p.Gln118*	0	0	No
*LMF1*	NM_022773.3	c.306G > A	p.Thr102=	0.4006	0.2796	rs3751667
		c.540G > A	p.Thr180=	0.2352	0.1899	rs2277892
		c.543G > A	p.Gly181=	0.3007	0.2776	rs2277893

### LPL mass and activity in post-heparin plasma of the proband

LPL mass and activity in the proband’s post-heparin plasma were measured in September 2017, when his TG level was 2.1 mmol/L (185.84 mg/dL). The LPL mass and activity were respectively 47% (160.7 U/L vs. 302 U/L) and 37% (0.053 vs. 0.143 mEq/L) of the mean values of 15 normal controls.

### In vitro analysis of the *LPL* p.Gln118* variant

**Fig. 4:**
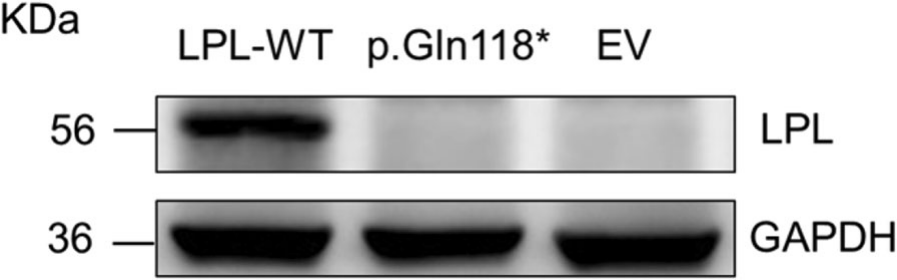
Functional characterization of the *LPL* p.Gln118* variant. Proteins prepared from the HEK293T cells that were transiently transfected with the *LPL* wild-type (WT) expression vector, the *LPL* p.Gln118* mutant expression vector and empty vector (EV), respectively, were used for Western blot analysis. GAPDH, loading control

*LPL* wild-type and p.Gln118* mutant expression plasmids were transiently transfected into HEK-293 T cells, respectively. LPL protein expression was analyzed by Western blot using transfected cell proteins and LPL activity was analyzed using transfected cell media. The LPL p.Gln118* mutant resulted in no detectable LPL activity and as shown in Fig. 4, the LPL p.Q118X resulted in undetectable LPL mass and activity, as compared to the positive results of *LPL* wild-type.

### Brief review of reported pathogenic *LPL* nonsense variants

Our literature search identified 18 papers that reported 12 distinct pathogenic *LPL* nonsense variants [26–43]. Variant zygosity, LPL mass and activity levels, TG level and disease outcomes in terms of HTG-AP were collected from each carrier whenever applicable (Table 2).

**Table 2:** Summary of the pathogenic *LPL* nonsense variants so far reported in the literature

Variant	Reference	Patient’s age	Country	Zygosity	The other variant in case of compound heterozygosity	LPL activity (% of normal)	LPL mass (% of normal)	TG level (mmol/L)	AP (times)
p.Trp14* (c.42G > A)	Nakamura et al. (1996) [33]	74	Japan	Homozygote		NI	0	18.5	No
	Li et al. (2018) [41]	61	China	Compound heterozygote	p.Leu279Val (c.835C > G)	39.9	48.60	38.6	Yes (3)
p.Cys54* (c.162C > A)	Chan et al. (2006) [37]	7 d	China	Compound heterozygote	p.Leu279Val (c.835C > G) homozygote	NI	NI	17.4	NI
p.Tyr88* (c.264 T > A)	Ebara et al. (2001) [36]	66	Japan	Homozygote		0	0	26.6	No
	Gotoda et al. (1991) [27]	3 m	Japan	Homozygote		14	0	216	No
	Gotoda et al. (1992) [28]	10 m	Japan	Compound heterozygote	p.Ala248LeufsTer4) (c.742del)	<5	0	46.2	NI
p.Trp91* (c.272G > A)	Sprecher et al. (1992) [30]	3	USA	Compound heterozygote	p.Ile221Thr (c.662 T > C)	0.0	34.2	> 226	Yes (recurrent)
		23	USA	Heterozygote		60.2	37.6	1.4	No
p.Tyr100* (c.300C > A)	Wilson et al. (1993) [31]	43	German and English-Irish ancestry	Compound heterozygote	p.Arg102Ser (c.306A > C)	6	28.5	16.7	Yes
p.Gln133* (c.397C > T)	Ishimura-Oka et al. (1992) [29]	1	English	Compound heterozygote	p.Trp113Arg (c.337 T > C)	22.4	NI	7.9	NI
	Emi et al. (1990) [26]	5 m	German and Polish ancestry	Homozygote		NI	NI	56.5	NI
p.Cys266* (c. 798C > A)	Takagi et al. (1994) [32]	54	Japan	Compound Heterozygote	p.Ser474*(p.1421C > G)	55.1	61.6	4.5	NI
p.Tyr289* (c.867C > A)	Evans et al. (2011) [39]	44	German	Compound heterozygote	p.Asp36Asn (c.106G > A)	NI	NI	11.6	NI
p.Cys291* (c.873C > A)	Jap et al. (2003) [43]	46	China	Compound heterozygote	p.Leu279Val(c.835C > G)	9	13	51.6	Yes (>10)
p.Tyr329* (c.987C > A)	Bertolini et al. (2000) [35]	7	Italy	Homozygote		0	0	29.8	Yes
	Hegele et al. (2018) [40]	NI	UK	Homozygote		0.1	NI	NI	NI
p.Trp409* (c.1227G > A)	Gotoda et al. (1991) [27]	6 m	Japan	Homozygote		15.6	5.4	67.9	NI
	Takagi et al. (1999) [34]	5 m	Japan	Compound heterozygote	p.Gly215Glu (c.644G > A)	<10	<10	48.1	No
	Suga et al. (1998) [42]	40	Japan	Homozygote		<1	<1	37.6	Yes
p.Trp421* (c.1262G > A)	Hooper et al. (2008) [38]	43	Australia	Compound heterozygote	p.Gly215Glu (c.644 G > A)	Very low	NI	32.2	Yes (recurrent)

*AP* Acute pancreatitis, *d* days, *m* Months, *LPL* Lipoprotein lipase, *NI* Not informative, *TG* Triglyceride

## Discussion

In this study, we reported a novel heterozygous nonsense variant in the *LPL* gene, p.Gln118* (c.351C > T), in one typical Chinese family with HTG-AP history. Presumably, this variant should cause a complete functional loss of the affected *LPL* allele due to its significant truncation of the 475 amino acid protein. Indeed, the LPL mass and activity in the proband’s plasma revealed a roughly 50% reduction as compared to normal controls. And in vitro, the results confirmed that the p.Gln118* mutant resulted in undetectable LPL protein and activity. Taken together, *LPL* p.Gln118* could be a novel and pathogenic *LPL* gene variant.

In this typical HTG-AP family, the proband, his mother and sister all had the *LPL* p.Gln118* nonsense variant, and separately had four, one and two times of HTG-AP onset. Moreover, HTG-AP was milder in the mother and older sister than the proband, as mild compared to moderate-severe. This variable clinical expression may be, at least partly, explained by the existence of an established secondary etiological factor, alcohol abuse, in the proband but not in the diseased mother and older sister. Although interplay between primary and secondary etiological factors in causing HTG-AP has been described in the literature [17, 18], to our best knowledge, the present study is the first to demonstrate the possible effect of alcohol abuse in modifying the clinical expression of a pathogenic genetic variant in the context of a HTG-AP family that exhibited a mode of monogenic inheritance. In this regard, it is pertinent to mention that, given the apparent effect of alcohol abuse in inducing or worsening HTG-AP, the proband has been required to quit alcohol since May 2017.

In this study, both in vivo and in vitro results showed that the *LPL* p.Gln118* nonsense variant could be pathogenic as resulting in complete functional loss of LPL mass and activity. Moreover, we briefly reviewed all the reported *LPL* nonsense variants together with the clinical features, as to evaluate the complex etiology of HTG or HTG-AP from a perspective of genotype and phenotype relationship. As shown in Table 2, the reported 12 *LPL* nonsense variants were detected in 20 subjects worldwide. As in detail, 8 were homozygotes, 2 were heterozygotes and 10 were compound heterozygotes. As expected, simple homozygotes showed no or barely detectable LPL mass and activity in the proband. The compound heterozygotes showed variable LPL mass and activity levels from zero to 50% of normal (depending upon the functional effect of the variant in trans). And, the heterozygotes showed LPL mass and activity levels that were around 50% of normal. We found correlation between mutation status and TG levels or occurrence of HTG-AP, as most informative adult patients had suffered recurrent AP with extreme high TG level, however, some patient is quite special. Taken p.Trp14* (c.42G > A) for example, the patient was had homozygote LPL variant, a 74-years-old Japanese with a complete LPL deficiency, never developed AP whilst the compound heterozygote, a 61-years-old Chinese whose LPL mass was ~ 50% of normal, had three times of AP (Table 1). Above all, the zygosity and AP occurrence of all the nonsense patients reported before emphasized again the complex etiology and expression of HTG-AP. Findings from this comparative analysis provide new evidence suggesting that new layers of complexity, beyond known genetic risk factors, predispose to, or prevent, the development of HTG-AP. Nowadays, there are some new genetic drugs for these LPL deficient patients like alipogene tiparvovec, LCQ908 etc., beyond the dietary management and usual pharmacologic therapies, these new genetic treatments can be certainly promising and effective therapy basing on the patients’ genetic background [44].

## Conclusion

To sum up, in this report, a novel LPL p.Gln118* (c.351C > T) variant was detected in one typical Chinese family with HTG-AP history, and had been verified to be pathogenic as resulting complete loss of LPL function in vitro. In particular, we highlighted a role of alcohol abuse in modifying the clinical expression of the disease in the proband. Moreover, we briefed reviewed all reported LPL nonsense variants, together with the phenotype, which may give new insights into the complex etiology of HTG-AP.

## Supplementary information

**Additional file 1: Figure S1.** Sanger sequencing electropherograms showing the three heterozygous *LMF1* synonymous variants detected in the proband.

## Data Availability

Data for the analyses are available from the corresponding author on request.

## Abbreviations

LPL: Lipoprotein lipase
TG: Triglyceride
HTG: Hypertriglyceridemia
HTG-AP: Hypertriglyceridemia-induced acute pancreatitis
FFA: Free fatty acid
HEK-293 T: Human embryonic kidney 293 T
AP: Acute pancreatitis
WB: Western blotting
APOC2: Apolipoprotein C-II
APOA5: Apolipoprotein A-V
LMF1: Lipase maturation factor 1
GPIHBP1: Glycosylphosphatidylinositol-anchored high density lipoprotein-binding protein 1
BMI: Body mass index
MSAP: Moderate severe acute pancreatitis
AKI: Acute kidney injury
ANC: Acute necrotic collection
TC: Total cholesterol
HDL: High density lipoprotein
LDL: Low density lipoprotein
PCR: Polymerase chain reaction
PHLA: Post-heparin lipase activity
BSA: Bovine serum albumin
HL: Hepatic lipase
EV: Empty vector
ATCC: American Tissue Culture Collection
DMEM: Dulbecco’s Modified Eagle’s Medium
FBS: Fetal Bovine Serum
PBS: Phosphate buffer saline
PI: Protease inhibitor
