# Supplementary material for: Identification of a novel LPL nonsense variant and further insights into the complex etiology and expression of hypertriglyceridemia-induced acute pancreatitis
Source: Lipids Health Dis. 2020 Apr 7;19:63. doi: 10.1186/s12944-020-01249-z (PMC7140582; doi:10.1186/s12944-020-01249-z)
Supplement: Supplementary file 1 — Additional file 1: Figure S1. Sanger sequencing electropherograms showing the three heterozygous LMF1 synonymous variants detected in the proband. [file 12944_2020_1249_MOESM1_ESM.docx]

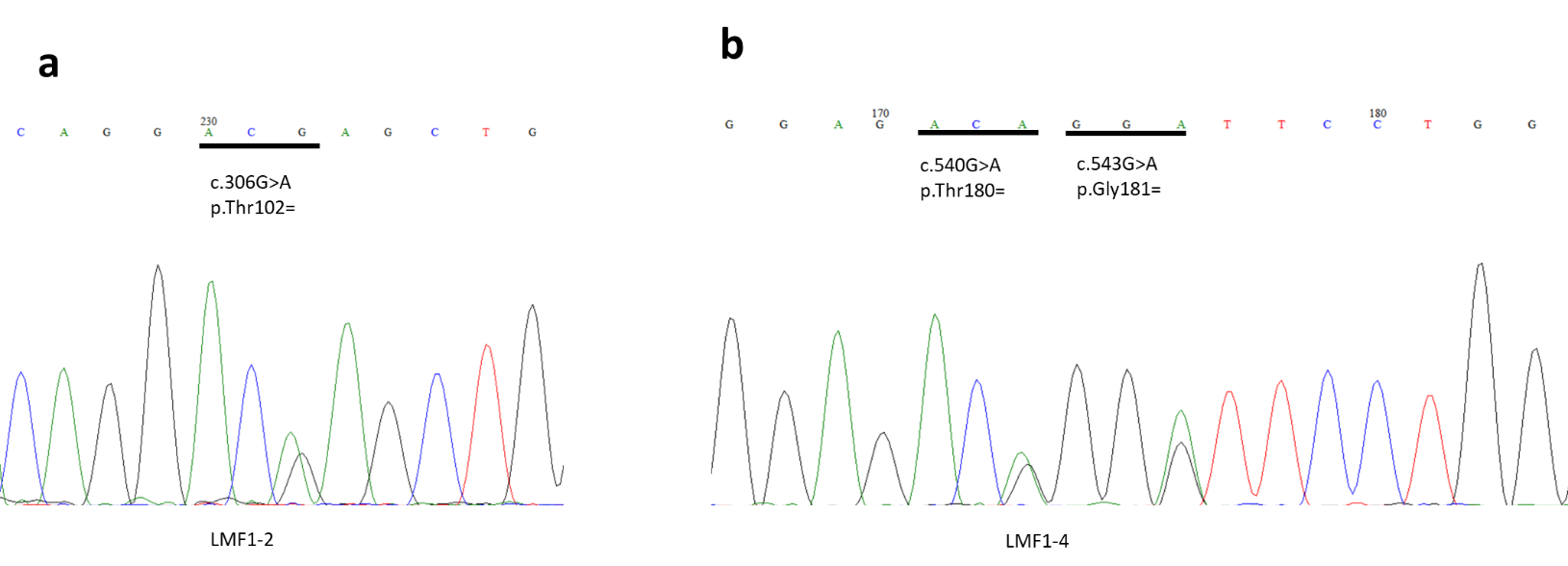


**Supplemental Figure S1.** Sanger sequencing electropherograms showing the three heterozygous *LMF1* synonymous variants detected in the proband.
